# Evaluation of Non-dietary Alternatives for Treatment of Adults With Celiac Disease

**DOI:** 10.3389/fnut.2020.562503

**Published:** 2020-10-19

**Authors:** Hugh James Freeman

**Affiliations:** Department of Medicine (Gastroenterology), University of British Columbia, Vancouver, BC, Canada

**Keywords:** sprue-like intestinal disease, celiac sprue, gluten-sensitive enteropathy (coeliac disease), gluten-free diet (GFD), celiac disease

## Introduction

Adult celiac disease (gluten-sensitive enteropathy, celiac sprue) is an immune-mediated mucosal disorder of the small intestine that occurs in genetically-susceptible individuals, often leading to diarrhea and weight loss. Usually, antibodies to tissue transglutaminase (as well as others) develop during the disease course. As a result, serological tests have been used as effective screening tools in many populations, particularly in directing biopsy testing to define prevalence of celiac disease. Diagnosis of untreated celiac disease in adults has traditionally been dependent upon a small intestinal biopsy showing the characteristic mucosal pathological changes that have been well-described elsewhere (1–4). Some of these features in untreated patients may include villous blunting, increased crypt length and mitotic activity, increased numbers of lamina propria lymphocytes and plasma cells, and finally, increased numbers of intraepithelial lymphocytes. These have also been classified in different ways, some emphasized to be more cumbersome than others (2, 3) to express the severity of the changes, such as mild, moderate, and severe (1, 4), primarily in the proximal small intestine (5). None of these pathological features are pathognomonic or specific for the diagnosis of untreated celiac disease, even the most severe changes. Indeed, celiac disease (celiac sprue, gluten-sensitive enteropathy) has been recognized for more than a half century by Dicke and his collegues (6) and others as a gluten-dependent disorder, and as emphasized elsewhere (7), a diagnosis of adult celiac disease depends on a critical further conformational step: *demonstration of a response to a strict gluten-free diet*.

## Implications for Treatment Trials

These criteria for diagnosis have important implications for clinical trials of any new or supplementary form of treatment, especially for a disease that is life-long. Most often, after a period of gluten-free diet treatment, resolution of clinical symptoms, readily quantified (e.g., diarrhea frequency, weight gain in kilograms, or pounds) and normalization of serological studies (i.e., levels quantified in International Units) occurs. However, improved symptoms and even normal serological results may not accurately reflect the presence or even the degree of persistent inflammatory mucosal changes in biopsies through the length of the intestinal tract. In future evaluations of any new proposed treatment, important endpoints to assess a positive treatment outcome should include changes from baseline (or the untreated state) in symptoms, serological results, and severity of mucosal biopsy changes. Ideally, this might include complete resolution of symptoms, normalization of serological studies and histopathological documentation of mucosal healing, including the proximal small intestinal mucosa (4, 5).

## Gluten-Free Diet Considerations

In general, a gluten-free diet is cumbersome, very costly, and difficult to follow precisely. Globally, even the definition of a gluten-free diet may not be well-defined or conceptually differs from one country to another. Some have proposed that for a food to be labeled “gluten-free,” it should contain no gluten, but others have a promoted a gluten-free product as up to 20 mg per kg of gluten (8). In some countries, sources for gluten-free products may be difficult to access and, often, gluten-free labeling is not provided in packaging. In some instances, the words “gluten-free” may be inaccurate and misleading since trace amounts of gluten may still be present. Most physicians, even expert gastroenterologists, do not have a precise knowledge of the gluten-content of most foods and, in many settings, a trained and interested dietitian is needed and essential to assist in compliance. Like any disorder, patients with celiac disease are also susceptible to the placebo and nocebo effects of any treatment, including a gluten- free diet (9). Increased costs related to a gluten-free diet may be covered in some countries, directly or indirectly by income tax concessions, but not all (10). To summarize, there remain many treatment issues for patients requiring a gluten-free diet. Some have used these issues to pursue an alternative form of therapy, but issues related to assessment of any novel form of treatment will remain, including added cost.

## Long-Term Effects of Gluten-Free Diets

The gluten-free diet is known to be highly effective in celiac disease. If compliant to a strict diet, the affected individual can anticipate a positive clinical result as well as a response in the abnormal changes in the small intestinal mucosa (11). Eventually, in the majority, complete histological recovery is possible. Gluten-free diets also have the potential, however, to cause some adverse effects (12), including development of nutrient deficiencies, obesity, particularly in children, and the risk of accumulation of several heavy metals (e.g., mercury, lead, cadmium) in urine and blood. This information may also be used as another reason for pursuing other non-dietary treatment options, even though the long-term effects, if any, of these observations are largely unknown.

## Assessment of Dietary Compliance

Dietary compliance and its assessment remains another significant issue (13). Evidence suggests that a failure of compliance leads to ongoing inflammatory mucosal change in the small bowel, causing an ongoing risk of complications related to only partially treated disease (e.g., osteopenia, malignancy). In general, it is believed that pre-school children are most easy to monitor because meal preparation and content remains under the parental control. Puberty and adolescence result in new challenges for the caregiver with loss of parental control and monitoring. In adults, compliance appears to be largely related to patient effort, education, and re-education provided largely by interested and knowledgeable physicians and specialized dietitians. Difficulties in clinical, serological, and histopathological assessment of compliance are evident.

### Clinical

Severe architectural changes may be present in asymptomatic patients, an indication that monitoring symptoms alone is likely to be inadequate as a measure of compliance (14). Patients with celiac disease neglected or lost to follow-up have increased rates of malignant and non-malignant complications. Celiac disease diagnosed later in life may also be associated with increased disease complications, including lymphoma, possibly reflecting persistent inflammatory change in the small intestinal mucosa for an extended period prior to diagnosis (15, 16).

### Serological

Serological assessment of compliance may also be highly misleading. In most, a strict gluten-free diet may result in a reduction in abnormally high antibody levels, often to a published normal serological range, and this was initially touted as evidence for dietary compliance. However, a second biopsy, even after serological studies had become normal, often revealed persistent inflammatory changes in the mucosa (17–21).

### Histological

Biopsy studies are more difficult and invasive, even in adults, but do provide very important information. The biopsy laboratory must have specific expertise in preparation, particularly orientation, of serially sectioned small biopsies for pathological interpretation (1, 22). Most clinicians have little training or interest in management of biopsies prior to submission to the biopsy laboratory and this is often reflected in the end result, a “garbage-in, garbage out” scenario. Trained observers are also an important asset. In a research environment that may be used to define the effects of a new treatment modality, biopsy interpretation is critical, and should be completed in a blinded fashion. This may become complicated if multiple readers are involved in biopsy processing. If multiple pathologists are involved, concordance between observers should be known along with the rate of intra-observer and inter-observer variation and a means of defining agreement, particularly, if interpretative differences exist on the degree of abnormality of biopsy abnormalities. In adults, complete healing of the mucosa may occur within 6 months, even if severe architectural disturbance was initially noted (11). However, for most, a more extended period is required. Over 80% of adult celiac disease patients on a strict gluten-free diet will show mucosal recovery and healing within 2 years, sometimes more, on a gluten-free diet (11). Regardless of the age range examined, adult women show higher rates of recovery than adult men while celiac disease patients diagnosed late in life have lower rates of healing. Stated differently, just as mucosal healing in response to a gluten-free diet has important confounding variables, non-dietary healing rates, if they occur, may also be time-, sex-, and age-dependent (11).

## Non-Responsive Celiac Disease

Normally, an adult with biopsy changes attributed to untreated celiac disease will be placed on a gluten-free diet and improvements in symptoms, serological results, and biopsy changes should result. Most often, difficulties in dietary compliance or ingestion of a ubiquitous or unrecognized gluten containing food source offer an explanation for failure of a prescribed gluten-free diet (i.e., classic example, communion wafers). Other entities that may cause similar histopathological changes should have been excluded (23). Currently, a special focus should be on an expanding list of infectious agents (Table 1) as well as pharmaceutical and biological agents (Table 2) that may either precipitate the appearance of celiac disease (if not previously recognized) or actually cause biopsy changes in the small intestinal mucosa that are indistinguishable from the changes of untreated celiac disease (24, 25). At present, the mechanisms involved in the development of these mucosal changes in both celiac disease and other disorders still requires elucidation. However, if a specific infectious agent or a medication and other immunotherapy agents) directly causes biopsy changes, a specific treatment or removal of the offending medications should be sufficient and not require a gluten-free diet or even proposed future potential alternative treatments.

**Table 1:** Sprue-like intestinal disease due to infectious agents.

**Viral agents**
Human immunodeficiency virus
Cytomegalovirus
**Protozoan**
Giardia lamblia
Isospora belli
Cryptosporidium parvum
Cytospora cayetanensis
Enterocytozoon bieneusi
**Bacteria**
Mycobacterium avium-intracellulare
Trophyrema whipplei
**Parasites**
Strongyloides stercoralis
Hookworm species
Schistosoma species
Capillaria species

**Table 2:** Sprue-like intestinal disease due to medications.

Non-steroidal anti-inflammatory drugs (NSAIDS, e.g., Sulindac)
Immunosuppressive agents (e.g., Azathioprine)
Anti-microbials (e.g., Neomycin)
Chemotherapeutic agents (e.g., Busulfan)
Vinca alkaloids (e.g., Vincristine)
Anti-metabolites (e.g., Methotrexate)
Angiotensin II receptor antagonists (e.g., Olmesartan)
Checkpoint inhibitors (e.g., Ipilimumab, Pembrolizumab, Nivolizumab)

Hypothetically, some celiac patients may be more “sensitive” to the offending peptides potentially resulting in rapid recurrence of pathological changes. Others may simply represent subgroups of celiac disease patients that are more resistant to treatment with a gluten-free diet, particularly the elderly. To date, there are no genetic markers known for possible celiac disease sub-types based on the time required or degree of treatment response to a gluten-free diet.

In general, the time course of all of these changes for individuals is not well-defined. Some have arbitrarily suggested a period of less than a year as sufficient to gauge the response of a gluten-free diet. This may be inadequate since several studies have demonstrated that while complete mucosal healing may occur within a few months, many patients require a longer duration of a gluten-free diet to document a defined histological treatment response, some beyond 1–2 years (11). If response is limited, or no histological response can be documented within a year, some have used the term “refractory celiac disease.” However, this duration of anticipated recovery may well-represent the non-specific nature of the inflammatory mucosal response to any injurious agent and the individual rate of healing that occurs in some celiac patients with removal of dietary gluten. These patients requiring a more prolonged period before precise documentation of celiac disease may pose a special problem in the assessment of celiac disease treatment with an alternative form of therapy.

## Studies of Novel Investigational Agents

These best studied to date descriptively fall into different classes (26–28), including endopeptidases (i.e., latiglutenase or ALV003, *Aspergillus niger* peptidase) and tight junction inhibitors (larazotide, or AT-1001). As noted (28), latiglutenase reduced gluten challenge-induced mucosal changes as well as increased numbers of intra-epithelial lymphocytes or had no effect on improving histologic scores or symptoms compared to placebo, while *Aspergillus niger* peptidase was not effective in preventing mucosal damage induced by 7 g gluten daily for 2 weeks. Larazotide reduced symptoms provoked by gluten challenge in celiac disease but had no impact on the primary outcome measure of intestinal permeability. A study using a *Bifidobacterium infantis* probiotic improved symptoms and lowered tTG antibodies, but failed to produce a significant effect on intestinal permeability measures. Some of these studies with these novel agents are proceeding to phase 3 trials with active recruitment of celiac disease patients.

Other studies differing descriptively in class and promote the use of gluten-sequestering polymers, transglutaminase enzyme inhibitors, and immune cell-targeted treatment including anti-IL-15 monoclonal antibodies, CCR9 antagonists, and integral antagonists, including vedolizumab, but early phase clinical trial results for all of these agents are still needed.

## Summary

For most patients with celiac disease, a gluten-free diet has been effective with few adverse effects. More importantly, these patients will usually have a detailed personal experience associated with a long history of use. To replace this approach with an alternative diet or supplement may be difficult. Ultimately, this approach may only offer a supplementary form of treatment to a patient on a well-established gluten-free diet. The principal issues then would be to provide an added form of low-cost treatment that provides added efficacy and has few adverse effects over the long term. Further studies may yield a treatment helpful to celiac disease patients.

## Author Contributions

The author confirms being the sole contributor of this work and has approved it for publication.

## Conflict of Interest

The author declares that the research was conducted in the absence of any commercial or financial relationships that could be construed as a potential conflict of interest.
